# Development of a Trait‐Based Risk Assessment to Minimise the Impacts of Trout on New Zealand Native Fishes

**DOI:** 10.1002/ece3.72480

**Published:** 2025-12-26

**Authors:** Ami Coughlan, Adam D. Canning, Russell G. Death

**Affiliations:** ^1^ School of Natural Sciences – Ecology Massey University Palmerston North New Zealand; ^2^ Wellington Fish and Game Council Palmerston North New Zealand; ^3^ Centre for Tropical Water and Aquatic Ecosystem Research (TropWATER) James Cook University Townsville Queensland Australia; ^4^ River Research Pohangina New Zealand

**Keywords:** fish conservation, introduced species management, New Zealand, *Oncorhynchus mykiss*, risk assessment, *Salmo trutta*

## Abstract

The management of introduced species to protect native fauna in the face of their rapid decline often presents a costly, technically complex, and sometimes socially controversial challenge. Prioritising when and where conservation efforts are best applied for the greatest efficacy is difficult. Using a systematic risk assessment framework effectively prioritises options among the range of management alternatives. Tensions exist between managing trout in New Zealand as a highly valued sport fishery and conserving native freshwater fish populations. We developed a trait‐based risk assessment matrix to identify the native fish most vulnerable to trout pressures and prioritise river reaches where those species are abundant for conservation focus. The trait‐based assessment indicated that all species identified as highly vulnerable to trout pressures were either non‐migratory galaxiids or mudfish. Trout overlapped in occurrence with approximately 10% of the entire extent of species deemed high risk to trout pressures, equating to 1626 km of river length, largely in central Otago and Hawkes Bay. While 1626 km of river is small compared to the entire riverine network (0.4%), it is still a substantial area for resource conservation interventions. Improving habitat quality to bolster native fish resilience to trout pressures, removing or reducing trout abundance are, however, potentially effective options. The approach adopted here will allow conservation managers to direct these actions to areas where they result in the greatest likelihood of positive outcomes.

## Graphical abstract

A trait‐based risk assessment framework was developed to identify New Zealand's native freshwater fish most vulnerable to the pressures of valued sports fisheries (trout). We further examine where the distributions between at‐risk species and trout are likely to overlap, providing priority locations for conservation managers to investigate further and intervene if needed. Trout overlapped in occurrence with approximately 10% of the entire extent of the most vulnerable species.

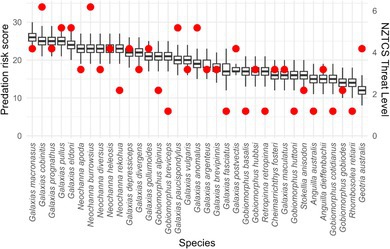

## 1 Introduction

Managing introduced species is challenging, costly, and often contentious because of conflicting values among stakeholders (Cuthbert et al. 2022; Gallardo et al. 2016; Vilà et al. 2011). Habitat destruction and climate change further exacerbate the impacts of introduced species (Atwater et al. 2017; Beaury et al. 2020). Estévez et al. (2015) found that 82% of conflicts over management stem from value disagreements, such as debates over wild horse control in the USA, Canada, Australia, and New Zealand. For example, the aerial culling of 600 wild horses in Australia's Guy Fawkes National Park, undertaken without community consultation, triggered widespread backlash and a ban on aerial culling (Chapple 2005). Management plans for introduced species that are primarily justified by ill‐informed environmental ideologies, such as an assumption that introduced species are always undesirable, can often be poorly attuned to the complexities of their local social landscapes (Crowley et al. 2017; Prévot‐Julliard et al. 2011). For long‐term success, introduced species management must be financially sustainable, ecologically effective, and socially acceptable (Burnett et al. 2023; Schlaepfer et al. 2011; van Eeden et al. 2020). Financial viability depends on secure, long‐term funding and the scale of management required. Key ecological questions include the impact thresholds of introduced species, differences from native species at similar densities, factors driving invasion time lags, and management needed to reach target population sizes (Havel et al. 2015).

New Zealand conservation managers face a similar challenge of managing introduced species, complicated by limited funding, politically driven policies, and competing values (Goldson et al. 2015; Peltzer et al. 2019; Tadaki et al. 2022). These species range from universally undesired pests (e.g., rats, mustelids, possums, wasps) to valued resources (e.g., cats, dogs, deer, pigs) and legally protected species (e.g., salmonids, perch, ducks, swans, pheasants, quail). Even for undesired species, debate exists over effective management strategies. Critics of the Predator Free 2050 policy argue that reducing introduced rodents, marsupials, and mustelids is unachievable with current resources, lacks consideration of ecological consequences, and diverts attention from habitat restoration and biodiversity sanctuaries that improve population spill over to adjacent habitats and improved metapopulation management (Linklater and Steer 2018; Peltzer et al. 2019). Similar debates surround salmonids, with some advocating their removal to protect native fish and others favouring habitat improvements to benefit both native species and salmonids (Closs 2024; Jones and Closs 2018; Tadaki et al. 2022).

New Zealand's freshwater fish fauna faces a multitude of stressors including climate change, urban and agricultural intensification and expansion, exotic fish species, decreasing water quality and quantity and reducing freshwater fish habitat (Joy et al. 2019; Joy 2014). While many are cryptic, benthic and nocturnal, they now co‐occur with introduced trout in much of their former range. Studies show non‐diadromous species like Canterbury galaxias ( *Galaxias vulgaris* ) are often absent from reaches dominated by large trout, though they persist in sites with smaller trout or high disturbance (Jolly et al. 2024; McHugh et al. 2010; McIntosh 2022). Trout may also indirectly affect native fish via altered invertebrate prey communities, though such effects are context‐dependent (Olsson et al. 2006; Townsend and McIntosh 1996). Disentangling trout impacts from broader environmental degradation is difficult because of concurrent pressures, lack of baseline data, and habitat variability across study sites.

Risk assessment frameworks provide a systematic method for evaluating the ecological risks posed by novel species to native biodiversity, aiding in prioritising management actions (Probert et al. 2020). These assessments use numeric scores derived from key traits of species (Rowe and Wilding 2012). The Australian Weed Risk Assessment (Pheloung et al. 1999) is the most widely used framework, adapted for various regions, including New Zealand, the USA, and Japan (Gordon et al. 2008). Risk assessments for freshwater fish are increasingly common, developed for regions like the USA, Australia, and the UK. In New Zealand, such frameworks could triage native freshwater fish species at risk from trout pressures more effectively than the New Zealand Threat Classification System (NZTCS), which focuses on population size and range but does not account for predation vulnerability. A trait‐based assessment could prioritise species based on factors such as diel activity overlap, body size, recruitment potential, behaviour, disturbance resilience, food availability, and refugia (Joy and Death 2013; McIntosh et al. 2010). Subsequent analyses could identify overlap between vulnerable species and trout, assess local impact severity, and determine appropriate management interventions. Given these complexities, trait‐based risk assessment provides a structured and transparent tool to identify where trout pressures are most likely to have ecological consequences.

In the present study, we develop and use a trait‐based risk assessment matrix to assess and rank the risk of each native freshwater fish species experiencing population‐level stress from trout pressures in New Zealand rivers (Coughlan 2022). We examined how trait‐based risk scores aligned with existing threat classifications and used distribution models to identify overlap hotspots where trout may pose the greatest risk. Rather than reviewing trout impacts per se, we aimed to provide an ecologically grounded tool for prioritising management interventions based on species traits, independent of stakeholder or legislative frameworks (Jellyman et al. 2018; Jones and Closs 2018; Joy and Atkinson 2012).

### 1.1 Socio‐Political Context for Trout Management in New Zealand

Acclimatisation societies introduced various non‐native species to New Zealand, with brown trout ( *Salmo trutta* ), rainbow trout ( *Oncorhynchus mykiss* ), and Chinook salmon ( *Oncorhynchus tshawytscha* ) becoming well established, while others like brook char ( *Salvelinus fontinalis* ) survived in isolated areas. Trout fisheries were managed by acclimatisation societies until 1990, when Fish and Game Councils, established under the Conservation Act 1987, took over. These Councils operate a “user‐pays, user‐says” model, funded entirely by hunting and fishing licence sales, to manage, maintain, and enhance freshwater sport fisheries and game bird populations in the interest of anglers. While Fish and Game manage sport fish and game bird populations and regulations, habitat management is primarily the responsibility of local councils or landowners, making advocacy for freshwater ecosystems a key role. Funded solely by licence sales, Fish and Game Councils are guided by elected anglers and focus heavily on advocating for healthy freshwater ecosystems, especially as most trout populations are now self‐sustaining. Since 1990, trout have not been introduced into trout‐free areas. This model affords a level of freshwater advocacy that greatly surpasses other non‐governmental environmental advocacy organisations and greater independence from Government interference than the Department of Conservation, which is responsible for managing native fish. For instance, Fish and Game and their predecessors played a key role in securing 12 of New Zealand's 15 Water Conservation Orders, the highest protection status for waterbodies in the country. Additionally, licence revenue has supported the restoration of over 200 wetlands (Canning et al. 2021), surpassing the efforts of any other agency in New Zealand and significantly contributing to habitats for native fauna (Garrett‐Walker et al. 2020; Stewart et al. 2022). In addition to the sustenance provided by caught fish, New Zealand anglers have reported wellbeing benefits from trout fishing, including enhanced mental clarity, stress reduction, opportunities for solitude and reflection, connections with others and the environment, and physical health improvements through low‐impact exercise (Stewart et al. 2024). Clearly, large‐scale attempts to eradicate trout would require major legislative change and necessitate a considerable trade‐off in values. In conserving native fish, it remains debated whether eradicating trout to reduce direct impacts on native species is preferable, or whether supporting trout fisheries is more beneficial, given Fish and Game's advocacy for improved land and water management that promotes healthier freshwater ecosystems and may mitigate environmental impacts on native fish despite trout's trophic effects.

In contrast, native fish management by the Department of Conservation has been inconsistent, constrained by variable government funding and shifting political priorities (Joy and Canning 2020; Seabrook‐Davison et al. 2010). In 2019, the previous Labour‐led coalition government introduced legislation changes to bolster the protection and management of native fish and freshwater ecosystems, including the development of native freshwater fish management plans and stronger catchment management plans. The new native fish conservation legislation aimed to provide tools to the Department of Conservation to address key threats such as fish passage barriers, habitat loss, and noxious species, while supporting fisheries restoration and Treaty settlement programs. While the ‘Essential Freshwater’ policy reform programme aimed to stop further degradation of water quality and habitat, reverse past damage to achieve healthy ecosystems within a generation, and address water allocation issues fairly and efficiently (Joy 2022; Prickett and Joy 2024). However, the implementation of policy and plans remains constrained by funding and government agendas, with the recently elected National‐led coalition government proposing changes that are argued to weaken freshwater protections instead of increasing permissibility in natural resource use (Prickett and Joy 2024). At present, no legislative changes to the management of trout and native interactions have been proposed. Conservation managers may have to balance the management of both trout and native fish populations, and interventions will need to be targeted towards managing the locations where trout are most detrimental to native fish. An objective prioritisation approach will be needed to manage the impacts of trout if contrasting value systems are to be considered. At those locations, managers may employ strategies to protect native species, like the current use of exclusion barriers to protect isolated non‐migratory galaxiids in the South Island (Jolly et al. 2024).

## 2 Methods

### 2.1 Risk Assessment Framework

For the risk assessment framework, native fish were scored and ranked based on the biological traits each species had that would increase their vulnerability to trout pressures (Table 1). Native fish assessed included all resident species with distributions mapped by Crow et al. (2014). The overall impact of trout pressures on a given native fish population is determined by population dynamics, which are in turn governed by fecundity and frequency of spawning throughout a season and lifetime (Stevens et al. 2016). Rapid growth, early maturation, short life span, high fecundity, and widespread dispersal and distribution (r‐selected traits) allow for high population resilience to disturbance events (Winemiller 2005; Winemiller and Rose 1992). However, migratory and long‐lived, late maturing fish (K‐selected traits) are exposed to increased ontogenetic jeopardy because of their movements between differing habitats and increased time spent in vulnerable life stages (Arthington et al. 2016; Winemiller 2005).

**TABLE 1:** Risk assessment matrix used to indicate the vulnerability of New Zealand's native fish to introduced trout.

Mediating factors	Assessment	Score	Weighting	Example
Overlapping physical habitat with trout when co‐occurring within the same river reach (micro‐niche habitat proximity increases interaction likelihood)	No or rare overlap	1	1	Dwarf galaxiids (*Galaxias divergens*) score 2 out of a potential 3 with macro‐habitat overlaps with trout across a proportion of their wide range. Benthic adults utilise differing microhabitat from trout species, fry and juveniles are pelagic, increasing predation risk
	Intermittent overlap	2		
	Persistent overlap	3		
Diel activity patterns (activities at similar times as trout: e.g., crepuscular activity patterns increase the likelihood of interactions)	No or rare overlap	1	1	Dwarf galaxiids receive a 2 out of 3 as they are primarily diurnal—while trout have predominantly crepuscular feeding patterns they will feed during the day
	Intermittent overlap	2		
	Similar diel patterns to trout	3		
Diet similarities (increase potential for competitive interactions)	No or few similarities	1	1	A diet of terrestrial and benthic invertebrates is likely to increase the chance of interactions between dwarf galaxiids and trout, leading to the given score of 2 of a possible 3
	Similar (aquatic inverts)	2		
	Very similar (aquatic & terrestrial inverts/piscivorous)	3		
Fecundity & egg size (many small eggs aid population resilience by increasing the number of larvae)	Many	1	2	Dwarf galaxiids score 2 out of a possible 6, spawning moderate quantities of large eggs in two spawning peaks
	Few, small eggs	2		
	Few, large eggs	3		
Age at reproductive maturity (longer maturation time increases likelihood of individuals not surviving to breed)	1 year	1	1	Female dwarf galaxiids mature in the beginning of their second year of life, giving them their score of 2
	1–3 years	2		
	> 3 years	3		
Larval dispersal ability (source/sink repopulation potential, population replenishment and resilience)	Diadromous	1	2	Dwarf galaxiids score the highest possible risk score here of 6, because of their large fry's limited dispersal ability reducing the likelihood of population recruitment from any upstream populations
	Non‐diadromous, widespread dispersal	2		
	Non‐diadromous, limited dispersal	3		
	Declining	2		
	Naturally uncommon	2		
	Nationally vulnerable	2		
	Data deficient	2		
	Nationally endangered	3		
	Nationally critical	3		
Adult body length (smaller adults more easily predated)	> 12 cm	1	2	With a maximum length of 8 cm, dwarf galaxiids are in the highest risk bracket for their small size, with a score of 6
	8–12 cm	2		
	< 8 cm	3		

*Note:* The matrix consists of eight factors mediating the potential negative impact of trout. Score allocation ranged from 1 to 3, indicating no/low risk to high risk respectively. Highly influential factors were also double‐weighted. Also provided is an example of the scoring process using Dwarf galaxiids ( *Galaxias divergens* ; Appendix 1).

For each risk factor, species were assigned a score from 1 (low risk) to 3 (high risk), reflecting increasing vulnerability to trout pressures. Four of the eight risk factors—fecundity and egg size, age at maturity, threat status, and adult body size—were considered to have greater influence on population‐level risk and were therefore double‐weighted (Table 1). The total risk score per species was calculated as the weighted sum of all eight traits, with possible scores ranging from 10 to 30. For example, dwarf galaxiids scored 3 for both fecundity and body size, which were each double‐weighted (i.e., 3 × 2 = 6), contributing 12 to the total score. While not a formal systematic review, trait scoring was informed by a structured review of available literature for each species, including ecological studies, species accounts, and expert assessments. The same criteria were applied consistently across all species based on the available data. Full trait definitions, scoring thresholds, and justification for each species' scores are provided in Table 2 and Appendix 1, along with literature references for transparency and future adaptability.

**TABLE 2:** Risk assessment scoring of New Zealand's native fish to screen their vulnerability to impacts by introduced trout, as per the criteria and weightings described in Table 1.

Species	Risk factors and weightings								
	Overlapping mesohabitat	Diet similarities	Diel activity patterns	Fecundity and egg size	Age at maturity	Larval dispersal	Adult size	Score	Risk rating
	1	1	1	2	1	2	2		
Bignose galaxiid (*Galaxias macronasus*)	2	2	2	3	2	3	3	26	High
Dusky galaxiid ( *Galaxias pullus* )	2	2	2	3	3	3	2	25	High
Lowland longjaw galaxiid (*Galaxias cobinitis*)	2	2	2	3	1	3	3	25	High
Upland longjaw galaxiid ( *Galaxias prognathus* )	2	2	2	3	1	3	3	25	High
Eldon's galaxiid ( *Galaxias eldoni* )	2	2	2	3	2	3	2	24	High
Canterbury mudfish ( *Neochanna burrowsius* )	2	2	1	3	2	3	2	23	High
Brown mudfish ( *Neochanna apoda* )	2	2	1	3	2	3	2	23	High
Black mudfish ( *Neochanna diversus* )	2	2	1	3	2	3	2	23	High
Northland mudfish (*Neochanna heleosis*)	2	2	1	3	2	3	2	23	High
Chatham Island mudfish (*Neochanna rekohua*)	2	2	1	3	2	3	2	23	High
Taieri Flathead galaxiid ( *Galaxias depressiceps* )	2	2	2	2	2	3	2	22	High
Dwarf galaxiid ( *Galaxias divergens* )	2	2	2	1	2	3	3	22	High
Gollum galaxiid ( *Galaxias gollumoides* )	2	2	2	1	1	3	3	21	Moderate
Tarndale bully (*Gobiomorphus alpinus*)	2	2	2	1	1	3	3	21	Moderate
Upland bully ( *Gobiomorphus breviceps* )	2	2	2	3	1	2	2	21	Moderate
Canterbury galaxiid ( *Galaxias vulgaris* )	2	2	2	1	2	2	3	20	Moderate
Alpine galaxiid ( *Galaxias paucispondylus* )	2	2	2	1	2	3	2	20	Moderate
Roundhead galaxiid ( *Galaxias anomalus* )	2	2	1	1	2	3	2	19	Moderate
Koaro ( *Galaxias brevipinnis* )	3	3	2	1	2	1	2	18	Moderate
Giant kokopu ( *Galaxias argenteus* )	3	3	3	1	3	1	1	18	Moderate
Shortjaw kokopu ( *Galaxias postvectis* )	3	3	2	1	3	1	1	17	Minor
Bluegill bully ( *Gobiomorphus hubbsi* )	2	2	2	1	1	1	3	17	Minor
Banded kokopu ( *Galaxias fasciatus* )	3	3	2	1	3	1	1	17	Minor
Cran's bully ( *Gobiomorphus basalis* )	2	2	2	1	1	2	2	17	Minor
Common smelt ( *Retropinna retropinna* )	3	2	3	1	1	1	2	17	Minor
Inanga ( *Galaxias maculatus* )	3	2	2	1	1	1	2	16	Minor
Torrentfish ( *Cheimarrichthys fosteri* )	2	2	2	1	2	1	2	16	Minor
Stokell's smelt ( *Stokellia anisodon* )	3	1	3	1	1	1	2	16	Minor
Redfin bully ( *Gobiomorphus huttoni* )	2	2	2	1	2	1	2	16	Minor
Longfin eel ( *Anguilla dieffenbachii* )	2	3	1	1	3	1	1	15	Minor
Shortfin eel ( *Anguilla australis* )	2	3	1	1	3	1	1	15	Minor
Common bully ( *Gobiomorphus cotidianus* )	2	2	2	1	1	1	2	15	Minor
Giant bully (*Gobiomorphus gobiodes*)	2	2	2	1	2	1	1	14	Minor
Black flounder (*Rhombosolea retiarii*)	1	3	2	1	2	1	1	14	Minor
Pouched lamprey ( *Geotria australis* )	1	1	1	1	3	1	1	12	Minor

*Note:* Further discussion and literature to support scoring are provided in Appendix 1.

Given that both score allocation and risk factor weighting were influenced by author judgement, we used a Monte Carlo simulation to assess the uncertainty in species risk scores by randomly altering both the scores and weights in their calculation. Using R 4.3.3 (R Core Team 2024), 10,000 iterations were conducted in which 30 elements (either scores or weights) were randomly selected and altered: scores were adjusted by ±1, and weights of 2 were randomly reassigned to either 1 or 2. After each iteration, species scores were recalculated based on the modified values and plotted to illustrate the potential variation because of uncertainty in score and weight assignment. Using the BAMMtools package (Rabosky and Goldberg 2015), Jenks Breaks was used to split the median risk scores into three risk groups (high, moderate, and minor).

Linear regression was used to examine the relationship between the median trait‐based trout pressures risk scores derived here, following uncertainty analysis, and the existing New Zealand Threat Classification System (NZTCS) status (Dunn et al. 2018), The six threat status levels were assigned a value from 1 to 8, ranging from ‘Not Threatened’ to ‘Nationally Critical’ respectively.

### 2.2 Mapping Interaction Risk

Following risk assessment scoring, the overlap in distributions between trout and native fish, using predictions from Crow et al. (2014), was used to identify the locations and extent of river reach with at least one high‐risk native fish potentially interacting with trout. This allows prioritisation of conservation efforts to where the most at‐risk species are likely to be affected by trout. Crow et al. (2014) predict the spatial distributions of all native and introduced freshwater fish across the entire river network (Snelder et al. 2010), using random forest models built using a national fish database and a suite of environmental variables. The predictions from Crow et al. (2014) have also been shown to produce consistent predictions with a separate distribution modelling exercise in A Canning (2018). The latter study also predicted very little influence by trout on the native fish probability of occurrence when comparing contemporary assemblages with those predicted in human stressor‐free reference conditions. The network is an end‐to‐end hydrological network of all surface waterways across the nation, composed of over 590,000 river reaches that are, on average, approximately 700 m long and total 413,106 km (Snelder et al. 2010). While New Zealand has three species of trout, which may differ in the extent to which they impact native species given differences in body size and gape size, this study considered overlap with any trout species as equal, as trout body sizes vary substantially between river systems, and the body sizes of trout across all New Zealand rivers have not been documented.

## 3 Results

**FIGURE 1:**
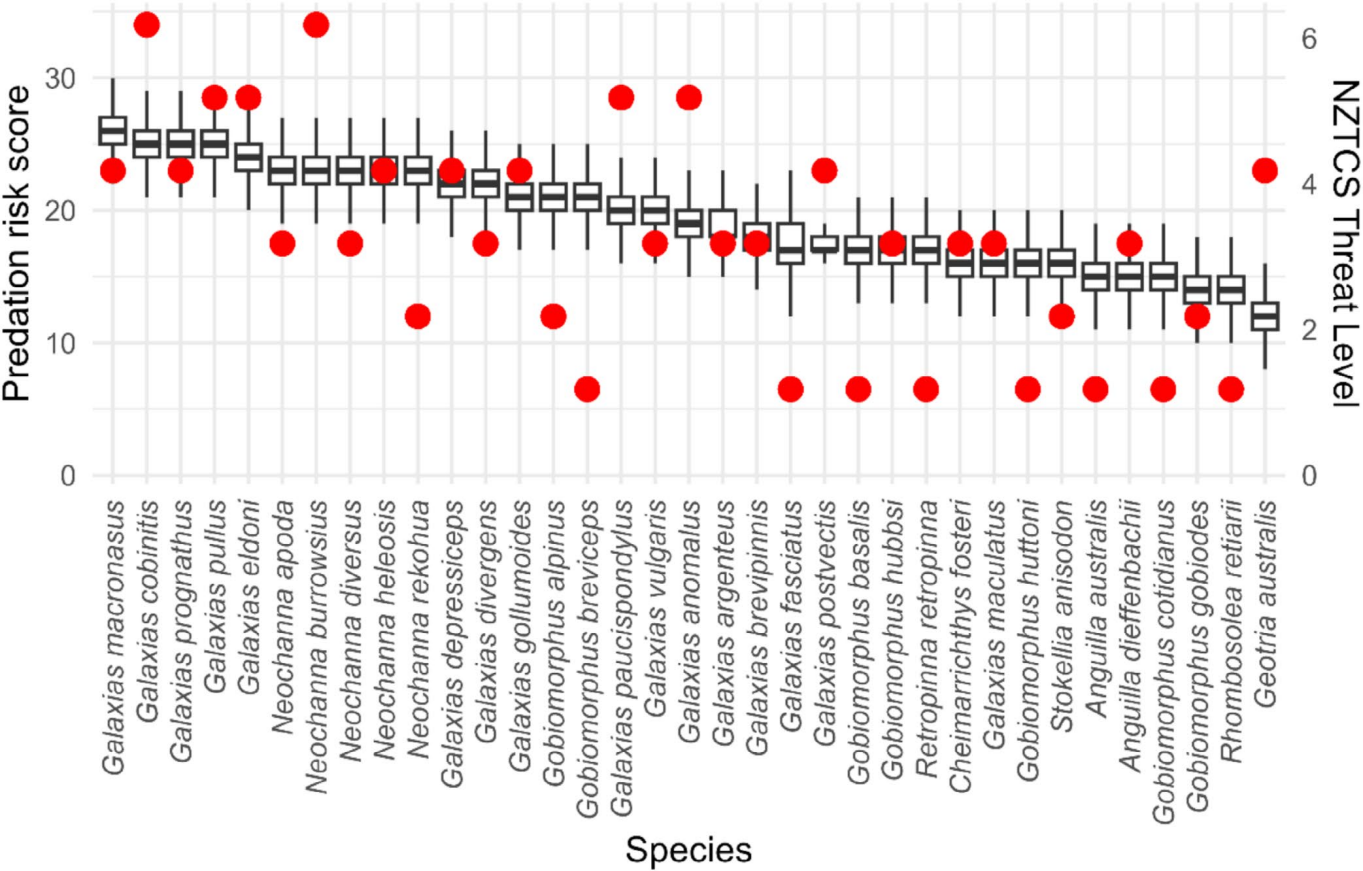
The distribution of species risk scores (boxplots) across 10,000 Monte Carlo simulations, where 30 elements (either scores or weights) were randomly modified in each iteration. Scores were adjusted by ±1, and weights of 2 were reassigned to either 1 or 2. Species are ordered by median score. The red dots range from 1 to 6 and correspond to NZTCS threat levels respectively: Not Threatened, Naturally Uncommon & Relict, Recovering, Declining, Nationally Vulnerable, Nationally Endangered, Nationally Critical.

The trait‐based assessment identified 12 species that are highly vulnerable and eight species that are moderately vulnerable to trout pressures. The most vulnerable species included several of the non‐diadromous galaxiids and the mudfish species, with the high‐risk rank position of these species also being largely retained in the uncertainty analysis simulated scenarios (Figure 1). The species deemed at least risk from trout predation included torrentfish, eels, bullies, smelt, flounder, and lamprey (Table 2). There was also a weak but positive linear relationship between the median trait‐based risk score and the NZCTS threat level (Adjusted *R* ^2^ = 0.29, *F*_1,33_ = 14.84, *p* < 0.001).

**FIGURE 2:**
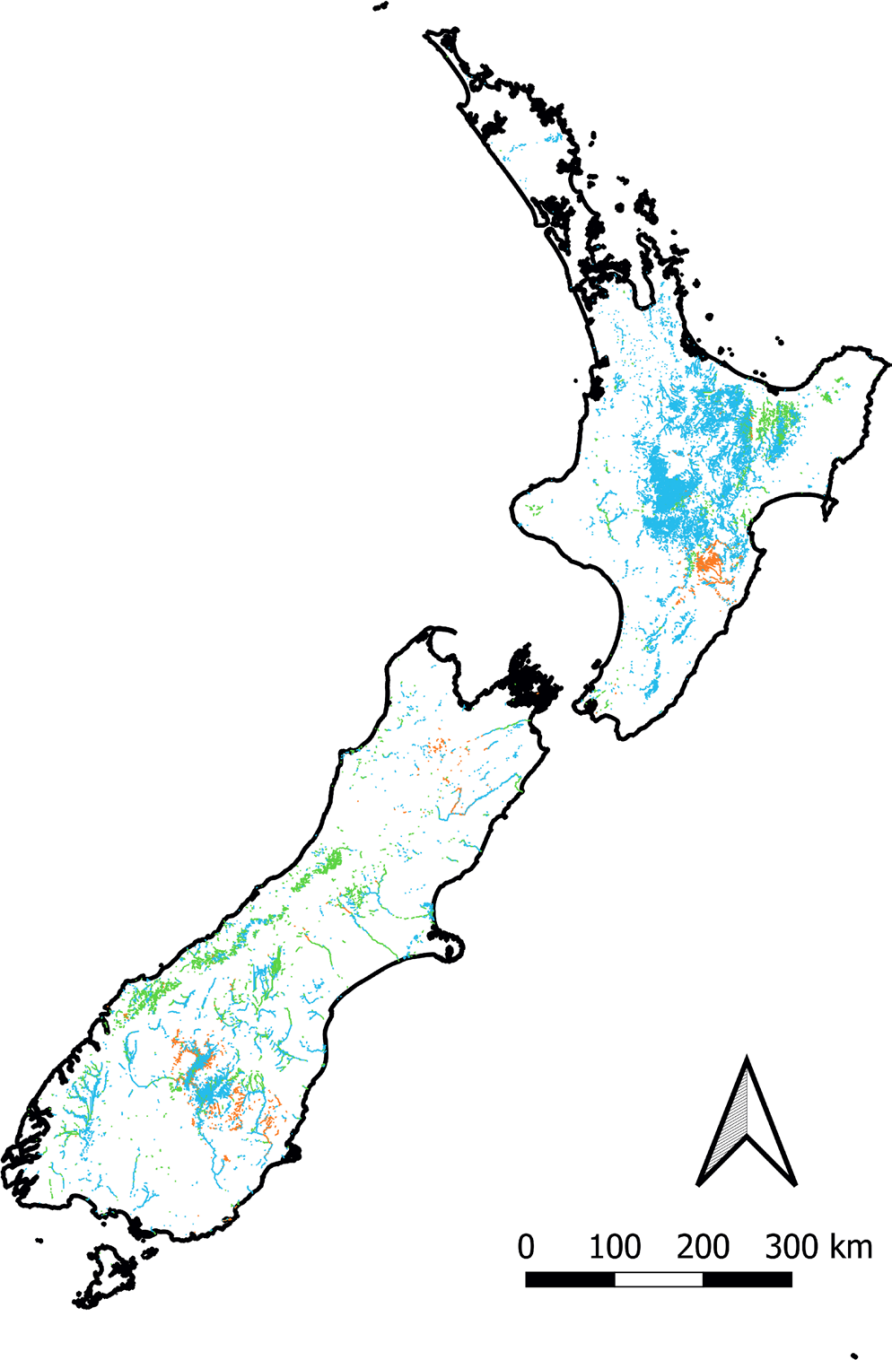
Map of New Zealand showing river reaches where native species are at high (orange), moderate (green), or low (blue) risk of negative population impacts because of trout pressures overlapping with trout presence. River reaches where there are no trout present are not shown.

Nationally, trout are predicted to overlap with at least one native fish species across a total stream length of 25,059 km (Figure 2). Of this overlapping distribution, 1626 km of reach contain both trout and at least one high‐risk native fish species (approximately 0.4% of all reaches nationally). Given that high‐risk native fish species are predicted to occupy approximately 16,179 km of river reach nationally, the distribution overlapping with trout accounts for approximately 10% of the entire range. Medium‐risk native fish species occupy ~60,072 km of river reaches nationally, with 5317.96 km (8.9%) of this overlapping with trout. Low‐risk native fish species occupy ~241,235 km of river reaches nationally, with 18,115 km (7.5%) of this overlapping with trout (Figure 2).

## 4 Discussion

Overall, the overlap between the ranges of native fish and trout was small, with approximately 10% of the entire extent of reaches with high‐risk species likely to overlap with the presence of trout. While the assessment involved risk score allocation by the authors, which introduces some subjectivity in literature interpretations and consequently classification of being at high risk, the Monte Carlo uncertainty analysis of score allocation indicated that variations in overall scores and rank positions were small. The distribution of detrimental impacts by trout, whether through competition or predation, is likely to be localised and may not be as widespread as previously suggested (Jellyman et al. 2018; McIntosh et al. 2010). The widespread decline of New Zealand's freshwater fish has been shown to be linked with changes in land use, with declining trends markedly worse at agricultural sites than those in native vegetation (Joy et al. 2019). Canning (2018) has also compared the current distribution of New Zealand's native freshwater fish with the expected distributions in the absence of human impact and found that differences in nutrient levels, presence of dams, and loss of natural riparian cover were the most influential factors. Trout only explained a small proportion of the absences, although this analysis only considered presence‐absence. That is not to say that trout do not have adverse effects on the distribution and abundance of native freshwater fish, but that other factors may also be important, such as widespread migration barriers, eutrophication, sedimentation, and highly degraded riparian areas. Resources and efforts should be prioritised to manage threats to freshwater fish proportional to their impact. Furthermore, habitat degradation and contraction may be exacerbating trout pressures by reducing population resilience and bringing trout in closer contact with native fish.

### 4.1 High Risk Interactions

Overall, mudfish and several non‐diadromous galaxiid species have populations most at risk to deleterious impacts from pressures, including from trout, and likely thus have a low resilience to disturbances more generally (Table 2). Mudfish have small, highly fragmented populations and are restricted in range because of ongoing habitat loss (McDowall 1990; West et al. 2015a, 2015b). While Canterbury mudfish have relatively higher fecundity levels, other mudfish species show low recruitment potential, and any loss to predation could have significant negative impacts on mudfish populations (Appendix 1; Eldon 1979a; O'Brien 2005; O'Brien and Dunn 2015). Dusky, lowland longjaw, Eldon's, bignose, upland longjaw, Taieri flathead, and dwarf galaxiids are all at high risk of negative impacts from trout pressures because of low recruitment rates, small adult size, and similarities in diet and habitat requirements (Allibone and Townsend 1997; McDowall 2006b). Figure 1 shows (in red) that trout overlap with high‐risk species is highly localised, rather than widespread nationally. These locations are primarily in the Otago region (non‐diadromous galaxiids) and Hawkes Bay (dwarf galaxiids).

### 4.2 Moderate Risk Interactions

Of the species with a moderate risk of deleterious impacts from trout, the roundhead and Gollum galaxiids and the Upland and Tarndale bullies were either classified with a highly vulnerable threat‐ranking or limited fecundity but had differing meso‐habitat preferences to trout (Appendix 1). The alpine and Canterbury galaxiids had similar meso‐habitat preferences but have high fecundity given they occur in generally unstable (regularly disturbed) rivers (Howard 2007; Woodford and McIntosh 2010, 2013). The larger diadromous galaxiid species (giant kokopu, shortjaw kokopu and koaro) are likely most vulnerable to trout pressures during their juvenile migrations as whitebait, while adults have habitat and diet preference overlaps with trout (Baker and Smith 2007; David et al. 2004; David et al. 2002). Moderate risk interactions were predicted to primarily occur around the central North Island and along the South Island's western ranges (Figure 1).

### 4.3 Low Risk Interactions

Longfin and shortfin eels, pouched lamprey, and black flounder are likely to be minimally affected by trout (i.e., at low risk) because of their size and differing feeding and habitat preferences (Appendix 1). Other low‐risk species, including inanga, Stokell's and common smelt, and common, redfin and Cran's bullies have traits conferring high resilience such as high fecundity and fast maturation, as well as wide dispersal and distribution and multiple spawning occasions in 1 year (Franklin, Ling, et al. 2015; McDowall 1990; Riddell 1982). Torrentfish, banded kokopu, and giant bully were also considered at low risk given their large adult size and the torrent habitat preferences of torrentfish, microhabitat preferences of banded kokopu, and the high fecundity of giant bully (McDowall 2006b, 2010).

### 4.4 Limitations and Research Needs

This risk assessment relied upon published literature to populate the assessment matrix; however, much of the research on New Zealand's native freshwater fish is limited. There are numerous fundamental knowledge gaps around biology, spawning, habitat requirements, and behaviours of many species (Dunn et al. 2018). While uncertainty analysis demonstrated scoring robustness to ranking, as research advances and informs knowledge gaps, or perspectives change, habitats change, or the health of native populations changes, so should this risk assessment with periodic review. Nonetheless, this assessment provides a transparent and systematic approach to indicate native fish risk of impact from trout pressures to facilitate prioritisation of conservation effort.

It is also unclear the extent to which trout, by means of both predation and competition, affect local native fish populations. While the risk of trout impacts on native species may be high in some locations, that does not necessarily mean the impact will also be high. The size of any deleterious impact will inevitably depend on numerous factors including the production rates of other prey fish and invertebrates, and the local trout population density. These in turn are all affected more generally by primary production, allochthonous input rates, nutrients, sediment, available habitats, migratory connectivity, temperature, and hydrological regime. The interaction of each factor in determining the impact of trout on native fish (or any trophic interaction for that matter) would be notoriously difficult to predict. Monitoring fish populations at high‐risk locations and responding accordingly to any low or declining populations would provide the most robust approach going forward. Although this study assessed current predicted overlap between trout and native species, the absence of overlap does not imply the absence of impact, particularly in cases where native fish may have already been extirpated from suitable habitats. Therefore, risk scores should be interpreted as indicative of species' vulnerability to trout pressures regardless of current co‐occurrence, and the absence of overlap should not preclude investigation.

While this study provides a trait‐based ecological risk assessment to inform conservation prioritisation, it did not include direct engagement with stakeholders. As such, it should be viewed as a technical tool to support, not prescribe, management action. Meaningful uptake will require future efforts to align ecological priorities with stakeholder values, particularly given the social, cultural, and recreational importance of trout. Participatory processes, including engagement with iwi, Fish and Game, anglers and local communities, could complement this ecological framework by identifying socially feasible management actions for high‐risk locations.

This study did not account for potential differences in impact severity between different trout species or spatiotemporal changes in trout populations. A global meta‐analysis by Korsu et al. (2010) highlights that impacts by brown trout are often more severe than those by rainbow trout, potentially owing to differences in adaptability, body and gape size, and aggression. However, trout abundance and body size vary substantially between river systems across all New Zealand rivers, with populations often fluctuating considerably, and this has not been documented for all rivers. An exercise that collates all trout abundance and size data from across the country to create a nationally comprehensive database and model could potentially be useful in applying risk weightings that are species and/or location specific. Furthermore, fisheries managers could carry out assessments of trout size and abundance at high and moderate risk sites and potentially weight sites accordingly.

### 4.5 Managing Pressures From Trout

There is a range of potential strategies for managing the pressure of trout on highly vulnerable native fish populations (Table 3). The trait‐based risk assessment provides a practical foundation for prioritising management actions. For example, species with high scores for habitat overlap and low dispersal—such as mudfish, lowland longjaw, and Eldon's galaxiids—are most suited to localised actions like trout exclusion barriers or protecting existing trout‐free refugia. In contrast, moderately vulnerable species with higher fecundity or broader habitat tolerance (e.g., alpine and Canterbury galaxiids) may benefit more from habitat improvement measures that increase resilience to disturbance, such as riparian restoration or sediment reduction. The assessment also highlights species where improving migratory connectivity must be carefully weighed against the risk of trout incursion (e.g., shortjaw kōkopu). These examples demonstrate how the framework can guide the type, location, and urgency of intervention by linking species traits to relevant stressors and likely benefit from specific actions. The risk assessment results therefore provide more than just a ranking—they underpin management triage. By combining trait‐based vulnerability scores with predicted spatial overlap, the assessment identifies where and for which species trout pressures are most likely to have population‐level effects. This directly informs the choice and prioritisation of the mitigation strategies presented in Table 3, ensuring that interventions are both ecologically justified and targeted to the specific risks faced by each native fish group. Two broad strategy approaches exist: (1) preventing and reducing the presence of trout around native fish, and (2) increasing the resilience of native fish to disturbances (such as trout pressures) by improving habitat quality (Table 3; Pingram et al. 2021). Although trout are generally absent from degraded sites due to their preference for cool, well‐oxygenated, low‐nutrient waters, they can still exert strong predation or competition pressures in otherwise high‐quality habitats. Habitat improvement in this context is not aimed at mitigating habitat degradation caused by trout, but at enhancing native fish resilience to trout‐related pressures and other cumulative stressors.

**TABLE 3:** Actionable management strategies to reduce the impact of trout predation on New Zealand's native freshwater fish.

Mitigation actions	Rationale
Minimise water abstractions to reduce very low flows and retain flood frequency and magnitude. For example, in the Manuherikia River, New Zealand, Leprieur et al. (2006) showed that more natural hydrological regimes benefitted Roundhead galaxias while reducing brown trout populations Local relationships between trout populations and flood frequency and magnitude could be derived to identify critical thresholds	Floods and very low flows tend to limit trout populations, instances where floods are reduced because of water abstractions may result in increased trout populations that consequently have greater predation pressure on native fish. Many native fish also rely on floods to signal key spawning events. Very low flows may increase interaction occurrence between trout and native fish (Bergerot and Cattanéo 2017; Young et al. 2010)
Promote the retention and enhancement of instream habitat diversity, particularly habitats that support the reproduction and refuge for highly vulnerable native species. In New Zealand, restoring the vast areas of lost and highly degraded spawning habitat and reducing deposited fine sediment should be a focus, with appropriate restoration targets developed. Sediment targets could be informed by Clapcott et al. (2011) and Franklin et al. (2019). Better understanding the habitat requirements of the highly vulnerable species should be a priority, followed by assessment of habitat availability and restoration options	Native fish habitat and enhancement may facilitate cohabitation by supporting more resilient populations that can better recover from multiple stressors. Refuge habitat may allow native fish to reduce exposure to predation. Given that many of NZ's species are benthic, reducing deposited fine sediment to increase interstitial spaces between gravels would support refuge, as well as increase invertebrate abundance, which may then reduce competition pressures between trout and native species. For example, Ramezani et al. (2014) found experimental removal of deposited fine sediment in NZ's Taieri River benefited invertebrate, native fish, and trout—highlighting a potential ‘win‐win’ scenario. While Hickford and Schiel (2014) showed that experimental livestock removal from *G. maculatus* spawning habitats resulted in a 400‐fold increase in egg density and a doubling in egg survival. Critical given that over 90% of New Zealand's wetlands, many of which would have supported spawning, has been drained
Advocate for minimised inputs of nutrients and pollutants from any source. In New Zealand, instream nutrient targets that account for differences in river morphology could be informed by Snelder et al. (2019) and Canning and Death (2023) whom examined relationships between nutrient concentrations and periphyton and macroinvertebrates, respectively	Nutrient inputs can directly increase algal and microbial production, causing complex cascading effects. These include promoting small invertebrates with high nutrient demands, which are less energetically rewarding for fish, and creating hypoxic conditions that affect organic matter availability and ecosystem functions. Excess nutrients can also disrupt metabolic pathways, harm aquatic organisms, exacerbate parasite infections, and reduce greenhouse gas mitigation. To mitigate these broad ecological impacts, limits on nutrient enrichment in rivers are essential (Dodds and Smith 2016). Metal and chemical pollutants impair fish species greatly decreasing predator avoidance ability (Thomas et al. 2016; Weis and Candelmo 2012)
Develop a tool to identify source populations, which sustain recruitment, and sink populations, which rely on immigration. Conservation efforts should prioritise protecting source populations and enhancing recruitment in sink areas	Sink populations experience a net loss of individuals and rely on immigration from healthier source populations to persist. This dependence makes them highly vulnerable to extirpation from threats such as trout predation or habitat degradation. Understanding the dynamics of source and sink populations is crucial for conservation, as protecting and maintaining source populations can bolster overall species resilience, while targeted management can reduce pressures on vulnerable sink populations. This knowledge informs prioritisation of conservation actions to sustain population networks and mitigate species declines (Lee and Perry 2019; Woodford and McIntosh 2011)
Seek to remove or remediate fish passage barriers, like perched culverts and dams, to maximise upstream habitat and access to spawning areas for migratory fish. However, barriers should not be removed where they are protecting upstream non‐migratory species from downstream trout populations	Improving fish passage allows migratory species to reach essential habitats for spawning and growth, supporting population resilience and ecosystem connectivity. However, barriers can act as important refuges for non‐migratory native fish by preventing trout access to upstream habitats where these vulnerable populations thrive. Balancing fish passage improvements with the need to protect isolated native fish populations ensures that conservation actions do not unintentionally harm species at risk. Previous analysis predicted that major migratory barriers explained more than 20% of the observed/expected ratio comparing species richness observed under contemporary conditions versus that expected under reference conditions (Canning 2018; Franklin and Gee 2019; McDowall 2006a)
Advocate for appropriate riparian vegetation, prioritising in places where riparian habitat is utilised by highly vulnerable native fish, where vegetation can effectively reduce nutrients and sediment, and along small streams where shading could mitigate rising water temperatures under future climates	Riparian vegetation stabilises stream banks, reduces runoff, and provides shelter and food inputs that enhance habitat complexity and support inter‐species coexistence. Shading by riparian plants helps regulate water temperatures, which is critical for the survival of temperature‐sensitive fish species. Targeting restoration in such areas maximises conservation outcomes and mitigates environmental impacts (Hickford and Schiel 2011; McKergow et al. 2016; Meijer et al. 2021)
Reduce trout populations in areas of high overlap with vulnerable native species using sustained removal efforts and/or exclusion barriers. Where appropriate, implement higher bag limits to support lower trout densities in key locations	Lower trout densities can reduce predation and competition pressure on native fish. Increased bag limits or exclusion zones may help maintain reduced populations in critical habitats (Klemetsen et al. 2003; McIntosh 2000). Exclusion barriers are particularly suitable for protecting non‐migratory native species, though potential impacts on migratory fauna must be considered (Jolly et al. 2024)

Preventing and reducing the presence of trout around native fish can include restricting the release of trout into new areas, reducing trout populations, installing migration barriers to limit the spread of trout into certain areas with non‐migratory native fish (Jolly et al. 2024; Novinger and Rahel 2003), and ensuring rivers retain natural flood frequency and severity (Bergerot and Cattanéo 2017; Gouraud et al. 2008). When reducing trout populations, managers need to be mindful that population reductions do not simply ease resource‐limited growth constraints and result in higher trout production rates—a phenomenon often used in fisheries to maximise sustainable harvest rates (Rytwinski et al. 2019; Vincenzi et al. 2008). For example, removal efforts in the Logan River, Utah, observed reduced density‐dependent mortality in the uncaught population (Saunders et al. 2015). Density‐dependent populations would require a higher level of sustained removal to overcome compensatory effects than density‐independent populations would—often where high temperatures, flooding and droughts limit population growth (Grossman and Simon 2020). Nonetheless, decisions around population reductions need to be weighed against the cultural importance of trout and the impacts on revenue from subsequent licence sales, which can be invested in advocacy and habitat restoration.

In addition to managing trout populations, improving native fish habitat more broadly—whether trout are present or absent—to bolster resilience to disturbances of any kind may be a more effective approach, particularly given widespread habitat degradation and the cumulative nature of disturbance impacts (Fraley et al. 2021; Pingram et al. 2021; Watson et al. 2021). Habitat improvements could include reducing water abstractions, reducing nutrient and sediment inputs, restoring riparian vegetation, restoring physical habitat, and restoring connectivity for migratory native fish (Table 3; Ouellet et al. 2022; Pelletier et al. 2020). While trout are generally sensitive to degraded environments and may benefit from habitat enhancements, native fish are also likely to benefit from improved spawning success, available refuge and availability of desirable macroinvertebrates. In trophic ecology, top‐down (or predator) controlled food webs with high emigration arise in instances where lower trophic levels have reduced ability to evade predation, reduced diversity, reduced competition for resources (such as desired macroinvertebrates), where autochthonous production dominates and disturbance events limit predators, while bottom‐up (or donor controlled) food webs occur in contrary conditions (Fronhofer et al. 2018; Gutgesell et al. 2024; Schulz et al. 2015). In New Zealand's Taieri River, an experiment removing deposited fine sediment improving benthic interstitial habitat potentially improved the density and composition of macroinvertebrates and native fish as well as trout. Canning and Death (2021) found that streams in New Zealand's Manawatu region with lower nutrient enrichment—where both trout and native fish co‐occurred—exhibited stronger trophic synergism (non‐obligatory positive feedbacks) than more nutrient‐enriched streams, illustrating how nutrient enrichment can weaken positive ecological feedbacks. Effectively, approaches to improve habitat aim to increase the pool of available resources rather than choosing winners and losers, or as the idiom goes “a rising tide lifts all boats.” Further research is needed to understand the habitat requirements of non‐migratory galaxiids, particularly in reaches where they coexist with trout, as well as the effectiveness of habitat restoration efforts. This would support more targeted and prioritised habitat enhancement actions. When improving migratory connectivity through the removal of barriers or installation of fish ladders, the potential for trout to move upstream into currently protected areas, either now or in the future with climate change, also needs to be kept in mind. Finally, Fish and Game's current policy not to liberate into new areas should be retained and could be expanded to include areas where highly vulnerable native fish reside.

Finally, an additional management lens involves accepting and managing some freshwater ecosystems as novel or hybrid systems, where introduced species such as trout have become entrenched and complete restoration to historical native assemblages is no longer feasible or desirable. In such cases, the focus may shift from eradicating non‐natives to maintaining ecological function, biodiversity, or cultural services (Hobbs et al. 2014; Miller and Bestelmeyer 2016). This approach is especially relevant where trout populations support valued recreational fisheries or where native species co‐adapt to altered conditions. A global meta‐analysis of brown trout introductions found that their negative ecological impacts tend to diminish over time, potentially because of native species adaptation or local extirpation, though impacts at the population level may persist (Závorka et al. 2018). While this trend should not be used to downplay trout impacts, it underscores the importance of context‐specific and temporally informed management strategies. Novel ecosystem thinking can help identify systems where managing for coexistence and ecosystem service provision is a pragmatic and socially acceptable outcome (Hallett et al. 2013; Simberloff 2015). Scenario‐based planning that weighs ecological risk, restoration potential, and stakeholder values is needed to guide such decisions (Woodford et al. 2016).

## 5 Conclusion

In summary, managing the impacts of introduced trout on native fish has, to date, been contentious given conflicting values and ideologies. To help manage the balance, this study uses a risk assessment approach to help prioritise river reaches for conservation intervention. Rivers with fish that were identified as highly vulnerable to trout largely occurred in the central Otago and Hawkes Bay regions. To manage these conflicts, we suggest an adaptive management approach beginning with in‐field assessment and monitoring along with the adoption of multiple strategies, potentially involving the reduction of trout populations and/or improvement in habitat. All species identified as highly vulnerable to trout pressures via the trait‐based assessment were either non‐migratory galaxiids or mudfish.

## Author Contributions

**Ami Coughlan:** conceptualization (equal), data curation (lead), formal analysis (lead), funding acquisition (supporting), investigation (lead), methodology (lead), project administration (equal), writing – original draft (lead). **Adam D. Canning:** data curation (supporting), formal analysis (lead), funding acquisition (lead), investigation (supporting), methodology (supporting), supervision (lead), visualization (equal), writing – original draft (supporting), writing – review and editing (equal). **Russell G. Death:** conceptualization (equal), data curation (supporting), formal analysis (supporting), funding acquisition (supporting), investigation (supporting), methodology (supporting), project administration (supporting), supervision (equal), writing – original draft (supporting), writing – review and editing (supporting).

## Conflicts of Interest

A.C. and A.D.C. were employed by the funder (Wellington Fish and Game Council), a statutory organisation which includes responsibility for the conservation and management of New Zealand's freshwater sports fisheries in accordance with the Conservation Act 1987 and the Freshwater Fisheries Regulations 1983.

## Data Availability

The manuscript contains all score allocations in Appendix 1. The original fish predictions were sourced from: https://dc.niwa.co.nz/niwa_dc/srv/api/records/d7d77b3b‐cd50‐4ee2‐adff‐1ca81d83b83e.

## Appendix 1

Trait‐based scoring matrix for native fish species. Each row represents an individual trait score for a given species, with justifications and supporting references. Traits marked with (×2) were double‐weighted in the final risk score. These scores underpin the trait‐based risk assessment of trout impacts.

**Table:**

Species	Scientific Name	Trait	Score	Justification	Citations
Bignose galaxiid	*Galaxias macronasus*	Habitat overlap	2	Restricted habitat, some overlap with trout reaches	Allibone et al. (2015b); Dunn et al. (2018); Jellyman et al. (2013); McDowall (1990); Sagar and Eldon (1983)
Bignose galaxiid	*Galaxias macronasus*	Diet similarity	2	Feeds on aquatic invertebrates; moderate overlap	
Bignose galaxiid	*Galaxias macronasus*	Diel activity	2	Day‐active and exposed to trout predation	
Bignose galaxiid	*Galaxias macronasus*	Fecundity & egg size (×2)	3	Low fecundity, large eggs	
Bignose galaxiid	*Galaxias macronasus*	Age at maturity (×2)	2	Matures at 2–3 years	
Bignose galaxiid	*Galaxias macronasus*	Dispersal/recruitment (×2)	3	Fragmented, non‐diadromous	
Bignose galaxiid	*Galaxias macronasus*	Adult body size (×2)	3	Small‐bodied; < 100 mm	
Dusky galaxiid	*Galaxias pullus*	Habitat overlap	2	Headwaters, some trout access	Allibone et al. (2015a); Closs et al. (2013); Dunn et al. (2018); Jellyman et al. (2013); McDowall (1990)
Dusky galaxiid	*Galaxias pullus*	Diet similarity	2	Aquatic inverts; moderate overlap	
Dusky galaxiid	*Galaxias pullus*	Diel activity	2	Likely active during daylight	
Dusky galaxiid	*Galaxias pullus*	Fecundity & egg size (×2)	3	Low fecundity, large eggs	
Dusky galaxiid	*Galaxias pullus*	Age at maturity (×2)	3	Matures > 3 years	
Dusky galaxiid	*Galaxias pullus*	Dispersal/recruitment (×2)	3	Highly restricted, isolated	
Dusky galaxiid	*Galaxias pullus*	Adult body size (×2)	2	Medium‐small; < 150 mm	
Lowland longjaw galaxiid	*Galaxias cobitinus*	Habitat overlap	2	Lowland spring‐fed habitats with trout	Allibone, West, Closs, et al. (2015); Baker et al. (2003); Dunn et al. (2018); Jellyman et al. (2013); McDowall and Waters (2002)
Lowland longjaw galaxiid	*Galaxias cobitinus*	Diet similarity	2	Benthic invertebrate diet; moderate overlap	
Lowland longjaw galaxiid	*Galaxias cobitinus*	Diel activity	2	Day‐active and exposed	
Lowland longjaw galaxiid	*Galaxias cobitinus*	Fecundity & egg size (×2)	3	Low fecundity, large eggs	
Lowland longjaw galaxiid	*Galaxias cobitinus*	Age at maturity (×2)	1	Matures young	
Lowland longjaw galaxiid	*Galaxias cobitinus*	Dispersal/recruitment (×2)	3	Fragmented habitat	
Lowland longjaw galaxiid	*Galaxias cobitinus*	Adult body size (×2)	3	Small‐bodied < 90 mm	
Upland longjaw galaxiid	*Galaxias prognathus*	Habitat overlap	2	Headwater streams with some trout access	Allibone et al. (2015a); Allibone et al. (2010); Bonnett (1992); Dunn et al. (2018); Howard (2014); Jellyman et al. (2013); McDowall (1990)
Upland longjaw galaxiid	*Galaxias prognathus*	Diet similarity	2	Invertebrate diet, moderate overlap	
Upland longjaw galaxiid	*Galaxias prognathus*	Diel activity	2	Likely active during the day	
Upland longjaw galaxiid	*Galaxias prognathus*	Fecundity & egg size (×2)	3	Very low fecundity	
Upland longjaw galaxiid	*Galaxias prognathus*	Age at maturity (×2)	1	Matures relatively young	
Upland longjaw galaxiid	*Galaxias prognathus*	Dispersal/recruitment (×2)	3	Landlocked and fragmented populations	
Upland longjaw galaxiid	*Galaxias prognathus*	Adult body size (×2)	3	Very small‐bodied	
Eldon's Galaxias	*Galaxias eldoni*	Habitat overlap	3	Confined to upper headwaters but trout introduced there	Allibone, Closs, Hitchmough, et al. (2015); Allibone and Townsend (1997); Dunn et al. (2018); Jellyman et al. (2013)
Eldon's Galaxias	*Galaxias eldoni*	Diet similarity	2	Aquatic invertebrates; moderate overlap	
Eldon's Galaxias	*Galaxias eldoni*	Diel activity	2	Day‐active; exposed to trout	
Eldon's Galaxias	*Galaxias eldoni*	Fecundity & egg size (×2)	3	Very low fecundity	
Eldon's Galaxias	*Galaxias eldoni*	Age at maturity (×2)	3	Late maturity ~4+ years	
Eldon's Galaxias	*Galaxias eldoni*	Dispersal/recruitment (×2)	3	No diadromy; isolated subpopulations	
Eldon's Galaxias	*Galaxias eldoni*	Adult body size (×2)	3	Small‐bodied; < 80 mm	
Canterbury Mudfish	*Neochanna burrowsius*	Habitat overlap	2	Found in modified lowland habitats; some overlap with trout habitat	Cadwallader (1975a, 1975b); Eldon (1979a, 1979b); O'Brien and Dunn (2007); West, Franklin, Crow, et al. (2015)
Canterbury Mudfish	*Neochanna burrowsius*	Diet similarity	2	Feeds on benthic invertebrates also consumed by trout	
Canterbury Mudfish	*Neochanna burrowsius*	Diel activity	1	Primarily nocturnal; reduced overlap with trout feeding periods	
Canterbury Mudfish	*Neochanna burrowsius*	Fecundity & egg size (×2)	3	High fecundity with many small eggs	
Canterbury Mudfish	*Neochanna burrowsius*	Age at maturity (×2)	2	Matures at 2 years	
Canterbury Mudfish	*Neochanna burrowsius*	Dispersal/recruitment (×2)	3	Non‐diadromous; fragmented and low dispersal potential	
Canterbury Mudfish	*Neochanna burrowsius*	Adult body size (×2)	2	Maximum ~175 mm body length	
Brown Mudfish	*Neochanna apoda*	Habitat overlap	2	Found in shallow wetlands and drains; some overlap with trout marginal habitats	Dunn et al. (2018); Eldon (1968); Nicholas Ling and Gleeson (2001); O'Brien and Dunn (2007); West, Franklin, Allibone, et al. (2015)
Brown Mudfish	*Neochanna apoda*	Diet similarity	2	Feeds on aquatic invertebrates; moderate dietary overlap with trout	
Brown Mudfish	*Neochanna apoda*	Diel activity	1	Nocturnal activity reduces overlap with trout feeding	
Brown Mudfish	*Neochanna apoda*	Fecundity & egg size (×2)	2	Moderate fecundity, larger eggs than some mudfish	
Brown Mudfish	*Neochanna apoda*	Age at maturity (×2)	2	Matures at 2 years	
Brown Mudfish	*Neochanna apoda*	Dispersal/recruitment (×2)	3	Non‐diadromous with fragmented distribution	
Brown Mudfish	*Neochanna apoda*	Adult body size (×2)	2	Adults reach ~140 mm	
Black Mudfish	*Neochanna diversus*	Habitat overlap	2	Found in temporary wetlands; potential marginal overlap	Closs et al. (2013); Dunn et al. (2018); Jellyman et al. (2013); Ling and Gleeson (2001); McDowall (2010); O'Brien and Dunn (2007); West et al. (2015b)
Black Mudfish	*Neochanna diversus*	Diet similarity	2	Invertebrate diet; moderate overlap	
Black Mudfish	*Neochanna diversus*	Diel activity	1	Nocturnal activity	
Black Mudfish	*Neochanna diversus*	Fecundity & egg size (×2)	2	Moderate fecundity with relatively large eggs	
Black Mudfish	*Neochanna diversus*	Age at maturity (×2)	2	Matures around 2 years	
Black Mudfish	*Neochanna diversus*	Dispersal/recruitment (×2)	3	Non‐diadromous; isolated populations	
Black Mudfish	*Neochanna diversus*	Adult body size (×2)	2	Adults grow up to 150 mm	
Northland Mudfish	*Neochanna heleios*	Habitat overlap	2	Lives in marginal wetlands, some overlap possible	Dunn et al. (2018); Jellyman et al. (2013); Ling and Gleeson (2001); McDowall (1990); O'Brien and Dunn (2007); West et al. (2015a)
Northland Mudfish	*Neochanna heleios*	Diet similarity	2	Invertebrate feeder with moderate overlap	
Northland Mudfish	*Neochanna heleios*	Diel activity	1	Primarily nocturnal	
Northland Mudfish	*Neochanna heleios*	Fecundity & egg size (×2)	2	Moderate fecundity with larger eggs	
Northland Mudfish	*Neochanna heleios*	Age at maturity (×2)	2	Matures at 2 years	
Northland Mudfish	*Neochanna heleios*	Dispersal/recruitment (×2)	3	Highly restricted distribution	
Northland Mudfish	*Neochanna heleios*	Adult body size (×2)	2	Adults ~130 mm	
Chatham Island Mudfish	*Neochanna rekohua*	Habitat overlap	1	No known overlap with trout habitats	Dunn et al. (2018); Jellyman et al. (2013); McDowall (1990); O'Brien and Dunn (2007)
Chatham Island Mudfish	*Neochanna rekohua*	Diet similarity	1	Limited data; unlikely overlap	
Chatham Island Mudfish	*Neochanna rekohua*	Diel activity	1	Likely nocturnal	
Chatham Island Mudfish	*Neochanna rekohua*	Fecundity & egg size (×2)	2	Likely similar to other mudfish	
Chatham Island Mudfish	*Neochanna rekohua*	Age at maturity (×2)	2	Estimated at 2 years	
Chatham Island Mudfish	*Neochanna rekohua*	Dispersal/recruitment (×2)	3	Naturally uncommon with low dispersal	
Chatham Island Mudfish	*Neochanna rekohua*	Adult body size (×2)	2	Adults ~150 mm	
Taieri Flathead galaxiid	*Galaxias depressiceps*	Habitat overlap	2	Overlap with trout in mid‐catchment Taieri	Allibone and Townsend (1997); Baker et al. (2003); Dunn et al. (2018); Jellyman et al. (2013); McDowall (1990); McDowall and Wallis (1996)
Taieri Flathead galaxiid	*Galaxias depressiceps*	Diet similarity	2	Aquatic invertebrates; some overlap	
Taieri Flathead galaxiid	*Galaxias depressiceps*	Diel activity	2	Likely active during trout feeding times	
Taieri Flathead galaxiid	*Galaxias depressiceps*	Fecundity & egg size (×2)	2	Moderate fecundity, moderate egg size	
Taieri Flathead galaxiid	*Galaxias depressiceps*	Age at maturity (×2)	2	Matures at 2–3 years	
Taieri Flathead galaxiid	*Galaxias depressiceps*	Dispersal/recruitment (×2)	3	Non‐diadromous, fragmented	
Taieri Flathead galaxiid	*Galaxias depressiceps*	Adult body size (×2)	2	Medium‐small	
Dwarf Galaxias	*Galaxias divergens*	Habitat overlap	2	Restricted to spring‐fed streams, some trout presence	Dunn et al. (2018); Hay (2009); Hopkins (1971); Jellyman et al. (2013); Jowett et al. (1996); McDowall (1990); West, Allibone, et al. (2015)
Dwarf Galaxias	*Galaxias divergens*	Diet similarity	2	Feeds on aquatic invertebrates; moderate overlap	
Dwarf Galaxias	*Galaxias divergens*	Diel activity	2	Day‐active and vulnerable to trout feeding	
Dwarf Galaxias	*Galaxias divergens*	Fecundity & egg size (×2)	3	Low fecundity, large eggs	
Dwarf Galaxias	*Galaxias divergens*	Age at maturity (×2)	2	Matures in 2nd year	
Dwarf Galaxias	*Galaxias divergens*	Dispersal/recruitment (×2)	3	Landlocked, fragmented populations	
Dwarf Galaxias	*Galaxias divergens*	Adult body size (×2)	3	Very small; adults ~65 mm	
Gollum galaxiid	*Galaxias gollumoides*	Habitat overlap	2	Confined to shaded bush streams with some trout presence	Allibone et al. (2015c); Dunn et al. (2018); Jellyman et al. (2013); McDowall (1990); McDowall and Chaddertoi (1999)
Gollum galaxiid	*Galaxias gollumoides*	Diet similarity	2	Feeds on benthic invertebrates	
Gollum galaxiid	*Galaxias gollumoides*	Diel activity	2	Crepuscular/nocturnal	
Gollum galaxiid	*Galaxias gollumoides*	Fecundity & egg size (×2)	1	Moderate fecundity	
Gollum galaxiid	*Galaxias gollumoides*	Age at maturity (×2)	1	Matures around 1–2 years	
Gollum galaxiid	*Galaxias gollumoides*	Dispersal/recruitment (×2)	3	Very limited dispersal	
Gollum galaxiid	*Galaxias gollumoides*	Adult body size (×2)	3	Very small‐bodied	
Tarndale bully	*Gobiomorphus alpinus*	Habitat overlap	2	High country streams with some trout access	Dunn et al. (2018); Jellyman et al. (2013); Ling, David, et al. (2015); McDowall (1990); McDowall and Stevens (2007); Smith et al. (2003)
Tarndale bully	*Gobiomorphus alpinus*	Diet similarity	2	Benthic invertebrates; moderate overlap	
Tarndale bully	*Gobiomorphus alpinus*	Diel activity	2	Likely diurnal	
Tarndale bully	*Gobiomorphus alpinus*	Fecundity & egg size (×2)	1	High fecundity	
Tarndale bully	*Gobiomorphus alpinus*	Age at maturity (×2)	1	Matures at 1–2 years	
Tarndale bully	*Gobiomorphus alpinus*	Dispersal/recruitment (×2)	3	Landlocked, limited recruitment	
Tarndale bully	*Gobiomorphus alpinus*	Adult body size (×2)	3	Small‐bodied, < 70 mm	
Upland Bully	*Gobiomorphus breviceps*	Habitat overlap	3	Widely overlaps in streams with trout	Cadwallader (1975a, 1975b); Dunn et al. (2018); Jellyman et al. (2013); Jowett and Boustead (2001); Jowett and Richardson (1995); McDowall (1990); West, David, Allibone, et al. (2015)
Upland Bully	*Gobiomorphus breviceps*	Diet similarity	2	Benthos feeders; moderate overlap with trout	
Upland Bully	*Gobiomorphus breviceps*	Diel activity	2	Some overlap in active periods	
Upland Bully	*Gobiomorphus breviceps*	Fecundity & egg size (×2)	2	Moderate fecundity, larger eggs	
Upland Bully	*Gobiomorphus breviceps*	Age at maturity (×2)	2	Matures in 1–2 years	
Upland Bully	*Gobiomorphus breviceps*	Dispersal/recruitment (×2)	1	Amphidromous; widespread dispersal	
Upland Bully	*Gobiomorphus breviceps*	Adult body size (×2)	2	Up to 90 mm; juveniles vulnerable	
Canterbury galaxiid	*Galaxias vulgaris*	Habitat overlap	2	Co‐occurs with trout in spring‐fed streams	Allibone et al. (2015b); Cadwallader (1973); Cadwallader (1975a, 1975b); Cadwallader (1976); Dunn et al. (2018); Glova et al. (1992); Jellyman et al. (2013); McDowall (1990)
Canterbury galaxiid	*Galaxias vulgaris*	Diet similarity	2	Feeds on aquatic invertebrates	
Canterbury galaxiid	*Galaxias vulgaris*	Diel activity	2	Day‐active; high exposure to trout	
Canterbury galaxiid	*Galaxias vulgaris*	Fecundity & egg size (×2)	1	Moderate fecundity	
Canterbury galaxiid	*Galaxias vulgaris*	Age at maturity (×2)	2	Matures at ~2 years	
Canterbury galaxiid	*Galaxias vulgaris*	Dispersal/recruitment (×2)	2	Fragmented populations	
Canterbury galaxiid	*Galaxias vulgaris*	Adult body size (×2)	3	Small‐bodied, < 100 mm	
Alpine galaxiid	*Galaxias paucispondylus*	Habitat overlap	2	Alpine streams above trout in many areas	Allibone et al. (2015a); Boddy and McIntosh (2017); Bonnett (1990); Bonnett (1992); Dunn et al. (2018); Jellyman et al. (2013); McDowall (1990); McIntosh (2000); Woodford and McIntosh (2013)
Alpine galaxiid	*Galaxias paucispondylus*	Diet similarity	2	Benthic invertebrates	
Alpine galaxiid	*Galaxias paucispondylus*	Diel activity	2	Day and crepuscular activity	
Alpine galaxiid	*Galaxias paucispondylus*	Fecundity & egg size (×2)	1	Relatively high fecundity	
Alpine galaxiid	*Galaxias paucispondylus*	Age at maturity (×2)	2	Matures at 2–3 years	
Alpine galaxiid	*Galaxias paucispondylus*	Dispersal/recruitment (×2)	3	Non‐diadromous; isolated populations	
Alpine galaxiid	*Galaxias paucispondylus*	Adult body size (×2)	2	Up to 140 mm	
Roundhead galaxiid	*Galaxias anomalus*	Habitat overlap	2	Headwater catchments, some trout access	Allibone, Ling, et al. (2015); Allibone and Townsend (1997); Baker et al. (2003); Dunn et al. (2018); Jellyman et al. (2013); McDowall (1990); McDowall and Wallis (1996); Moore et al. (1999)
Roundhead galaxiid	*Galaxias anomalus*	Diet similarity	2	Feeds on benthic invertebrates	
Roundhead galaxiid	*Galaxias anomalus*	Diel activity	1	Reduced overlap due to nocturnal habits	
Roundhead galaxiid	*Galaxias anomalus*	Fecundity & egg size (×2)	1	Relatively high fecundity	
Roundhead galaxiid	*Galaxias anomalus*	Age at maturity (×2)	2	Matures at ~2 years	
Roundhead galaxiid	*Galaxias anomalus*	Dispersal/recruitment (×2)	3	Non‐diadromous and restricted	
Roundhead galaxiid	*Galaxias anomalus*	Adult body size (×2)	2	Medium size; up to 110 mm	
Koaro	*Galaxias brevipinnis*	Habitat overlap	2	Riffles and boulder streams; some overlap with trout	Bell (2001); David et al. (2014); Dunn et al. (2018); Glova and Sagar (1989); Jellyman et al. (2013); Kusabs and Swales (1991); McDowall (1990); McEwan and Joy (2014a); McIntosh (2000); Rowe, Konui, and Christie (2002)
Koaro	*Galaxias brevipinnis*	Diet similarity	2	Invertebrate feeder; diet overlaps with trout	
Koaro	*Galaxias brevipinnis*	Diel activity	1	Diel feeding differences with trout	
Koaro	*Galaxias brevipinnis*	Fecundity & egg size (×2)	2	Moderate fecundity, small eggs	
Koaro	*Galaxias brevipinnis*	Age at maturity (×2)	2	Matures after 2+ years	
Koaro	*Galaxias brevipinnis*	Dispersal/recruitment (×2)	1	Diadromous; broad dispersal	
Koaro	*Galaxias brevipinnis*	Adult body size (×2)	2	Up to 270 mm; small juveniles vulnerable	
Giant Kokopu	*Galaxias argenteus*	Habitat overlap	3	Overlaps with trout in pools and slow‐flowing waters	Baker and Smith (2007); Bonnett and Lambert (2002); Bonnett and Sykes (2002); David (2003); David et al. (2004); Dunn et al. (2018); Franklin, Smith, et al. (2015); Hansen et al. (2004); Jellyman et al. (2013); McDowall (1990); West, David, Franklin, et al. (2015)
Giant Kokopu	*Galaxias argenteus*	Diet similarity	2	Predatory; generalist diet overlaps with trout	
Giant Kokopu	*Galaxias argenteus*	Diel activity	2	Activity periods overlap with trout	
Giant Kokopu	*Galaxias argenteus*	Fecundity & egg size (×2)	1	High fecundity with small eggs	
Giant Kokopu	*Galaxias argenteus*	Age at maturity (×2)	3	Matures at 3+ years; lives up to 30 years	
Giant Kokopu	*Galaxias argenteus*	Dispersal/recruitment (×2)	2	Some freshwater recruitment; partially landlocked	
Giant Kokopu	*Galaxias argenteus*	Adult body size (×2)	1	Large size reduces trout predation risk	
Shortjaw Kokopu	*Galaxias postvectis*	Habitat overlap	2	Small, clear forest streams; some overlap with trout	Allibone et al. (2003); Dunn et al. (2018); Goodman (2002); Jellyman et al. (2013); McDowall et al. (1996); McDowall (1990); McEwan and Joy (2014a); West, David, Hitchmough, et al. (2015)
Shortjaw Kokopu	*Galaxias postvectis*	Diet similarity	2	Feeds across the water column on inverts	
Shortjaw Kokopu	*Galaxias postvectis*	Diel activity	2	Nocturnal and crepuscular feeding	
Shortjaw Kokopu	*Galaxias postvectis*	Fecundity & egg size (×2)	2	Moderate fecundity; large eggs	
Shortjaw Kokopu	*Galaxias postvectis*	Age at maturity (×2)	2	Matures at 3+ years	
Shortjaw Kokopu	*Galaxias postvectis*	Dispersal/recruitment (×2)	2	Irregular recruitment; freshwater dependent	
Shortjaw Kokopu	*Galaxias postvectis*	Adult body size (×2)	2	Up to 350 mm; moderate trout vulnerability	
Bluegill bully	*Gobiomorphus hubbsi*	Habitat overlap	2	Turbulent riffle zones may co‐occur with trout	Allibone, David, Franklin, et al. (2015); Atkinson and Joy (2009); Dunn et al. (2018); Jarvis (2015); Jellyman et al. (2013); Jowett and Richardson (1995); Jowett et al. (1996); McDowall (1990)
Bluegill bully	*Gobiomorphus hubbsi*	Diet similarity	2	Inverts; some overlap	
Bluegill bully	*Gobiomorphus hubbsi*	Diel activity	2	Likely overlaps with trout feeding times	
Bluegill bully	*Gobiomorphus hubbsi*	Fecundity & egg size (×2)	1	High fecundity	
Bluegill bully	*Gobiomorphus hubbsi*	Age at maturity (×2)	1	Matures within 1 year	
Bluegill bully	*Gobiomorphus hubbsi*	Dispersal/recruitment (×2)	1	Amphidromous, widespread recruitment	
Bluegill bully	*Gobiomorphus hubbsi*	Adult body size (×2)	3	Small, < 70 mm	
Banded Kokopu	*Galaxias fasciatus*	Habitat overlap	2	Small streams with undercut banks may co‐occur with trout	David et al. (2019); Dunn et al. (2018); Jellyman et al. (2013); McCullough (n.d.); McCullough (2016); McDowall (1990); West, Crow, David, Ling, et al. (2015)
Banded Kokopu	*Galaxias fasciatus*	Diet similarity	2	Feeds on aquatic and terrestrial invertebrates like trout	
Banded Kokopu	*Galaxias fasciatus*	Diel activity	2	Territorial and crepuscular like trout	
Banded Kokopu	*Galaxias fasciatus*	Fecundity & egg size (×2)	1	High fecundity with small eggs	
Banded Kokopu	*Galaxias fasciatus*	Age at maturity (×2)	2	Matures at 2–4 years	
Banded Kokopu	*Galaxias fasciatus*	Dispersal/recruitment (×2)	1	Widespread and capable of dispersal	
Banded Kokopu	*Galaxias fasciatus*	Adult body size (×2)	2	Adults up to 300 mm	
Cran's bully	*Gobiomorphus basalis*	Habitat overlap	2	Lowland Canterbury streams; trout present	Dunn et al. (2018); Franklin, Ling, et al. (2015); Hicks and McCaughan (1997); Jellyman et al. (2013); McDowall (1990); Riddell (1982)
Cran's bully	*Gobiomorphus basalis*	Diet similarity	2	Benthic invertebrates; some overlap	
Cran's bully	*Gobiomorphus basalis*	Diel activity	2	Day‐active and exposed	
Cran's bully	*Gobiomorphus basalis*	Fecundity & egg size (×2)	1	Moderate fecundity	
Cran's bully	*Gobiomorphus basalis*	Age at maturity (×2)	1	Matures at 1 year	
Cran's bully	*Gobiomorphus basalis*	Dispersal/recruitment (×2)	2	Restricted to landlocked Canterbury populations	
Cran's bully	*Gobiomorphus basalis*	Adult body size (×2)	2	Typical size ~90 mm	
Common Smelt	*Retropinna retropinna*	Habitat overlap	2	Found in lowland rivers and lakes, often stocked with trout	Dunn et al. (2018); Franklin et al. (2014); Jellyman et al. (2013); McDowall (1990); Rowe (1984)
Common Smelt	*Retropinna retropinna*	Diet similarity	2	Omnivorous and pelagic, moderate overlap with trout diet	
Common Smelt	*Retropinna retropinna*	Diel activity	2	Day‐active; overlaps with trout feeding patterns	
Common Smelt	*Retropinna retropinna*	Fecundity & egg size (×2)	1	High fecundity, small eggs	
Common Smelt	*Retropinna retropinna*	Age at maturity (×2)	1	Matures within 1 year	
Common Smelt	*Retropinna retropinna*	Dispersal/recruitment (×2)	1	Diadromous; high dispersal	
Common Smelt	*Retropinna retropinna*	Adult body size (×2)	1	Adults ~125 mm	
Inanga	*Galaxias maculatus*	Habitat overlap	3	Drift‐feeds in slow pools, often co‐occurs with trout	David et al. (2019); David, West, et al. (2015); Dunn et al. (2018); Jellyman et al. (2013); Jowett (2002); McDowall (1990); Stevens et al. (2016); Yungnickel et al. (2020)
Inanga	*Galaxias maculatus*	Diet similarity	2	Omnivorous; feeds on invertebrates also targeted by trout	
Inanga	*Galaxias maculatus*	Diel activity	2	Pelagic and active during trout feeding times	
Inanga	*Galaxias maculatus*	Fecundity & egg size (×2)	1	Very high fecundity with small eggs	
Inanga	*Galaxias maculatus*	Age at maturity (×2)	1	Matures quickly; some die after the first spawn	
Inanga	*Galaxias maculatus*	Dispersal/recruitment (×2)	1	Highly dispersive; marine phase	
Inanga	*Galaxias maculatus*	Adult body size (×2)	1	Small; max ~110 mm	
Torrentfish	*Cheimarrichthys fosteri*	Habitat overlap	1	Prefer fast‐flowing riffles not commonly used by trout	Allibone, David, Franklin, Ling, et al. (2015); Dunn et al. (2018); Glova et al. (1987); Jellyman et al. (2013); Jowett et al. (1996); McDowall (1990); McDowall (2000); Scrimgeour and Eldon (1989); Warburton (2015)
Torrentfish	*Cheimarrichthys fosteri*	Diet similarity	2	Benthic invertebrates; moderate overlap with trout	
Torrentfish	*Cheimarrichthys fosteri*	Diel activity	1	Nocturnal; low overlap with trout feeding	
Torrentfish	*Cheimarrichthys fosteri*	Fecundity & egg size (×2)	1	High fecundity, small eggs	
Torrentfish	*Cheimarrichthys fosteri*	Age at maturity (×2)	2	Matures in 2–3 years	
Torrentfish	*Cheimarrichthys fosteri*	Dispersal/recruitment (×2)	1	Diadromous; wide dispersal	
Torrentfish	*Cheimarrichthys fosteri*	Adult body size (×2)	2	Adults up to 200 mm	
Stokell's smelt	*Stokellia anisodon*	Habitat overlap	3	Lowland lakes and rivers with trout	David, Crow, et al. (2015); Dunn et al. (2018); Jellyman et al. (2013); McDowall (1990); Ward et al. (2005)
Stokell's smelt	*Stokellia anisodon*	Diet similarity	1	Planktivorous; minimal overlap with trout	
Stokell's smelt	*Stokellia anisodon*	Diel activity	3	Schooling pelagic species; overlaps with trout	
Stokell's smelt	*Stokellia anisodon*	Fecundity & egg size (×2)	1	High fecundity	
Stokell's smelt	*Stokellia anisodon*	Age at maturity (×2)	1	Matures quickly, likely within a year	
Stokell's smelt	*Stokellia anisodon*	Dispersal/recruitment (×2)	1	Broad dispersal via lakes and estuaries	
Stokell's smelt	*Stokellia anisodon*	Adult body size (×2)	2	Adults ~100 mm	
Redfin Bully	*Gobiomorphus huttoni*	Habitat overlap	2	Steeper gradient and small streams; limited overlap	Dunn et al. (2018); Jellyman et al. (2013); Jowett et al. (2009); Ling, Allibone, et al. (2015); McDowall (1965); McDowall (1990); McEwan and Joy (2014b)
Redfin Bully	*Gobiomorphus huttoni*	Diet similarity	2	Feeds on benthic invertebrates	
Redfin Bully	*Gobiomorphus huttoni*	Diel activity	2	Active during the day	
Redfin Bully	*Gobiomorphus huttoni*	Fecundity & egg size (×2)	2	Moderate fecundity	
Redfin Bully	*Gobiomorphus huttoni*	Age at maturity (×2)	2	Matures at 1–2 years	
Redfin Bully	*Gobiomorphus huttoni*	Dispersal/recruitment (×2)	1	Widespread recruitment via the marine phase	
Redfin Bully	*Gobiomorphus huttoni*	Adult body size (×2)	2	Adults ~90–100 mm	
Longfin Eel	*Anguilla dieffenbachii*	Habitat overlap	2	Shares lowland and midland habitats with trout	Beentjes et al. (2006); Broad (2002); Cadwallader (1975a, 1975b); Dunn et al. (2018); Glova et al. (1998); Jellyman (2016); Jellyman (1996); Jellyman (2007); Jellyman et al. (2013); Jowett and Richardson (1995); McDowall (1990); Pike et al. (2015); Rowe, Smith, et al. (2002); Sagar et al. (2005)
Longfin Eel	*Anguilla dieffenbachii*	Diet similarity	2	Both are piscivorous and carnivorous	
Longfin Eel	*Anguilla dieffenbachii*	Diel activity	1	Primarily nocturnal; limited direct interaction	
Longfin Eel	*Anguilla dieffenbachii*	Fecundity & egg size (×2)	1	Extremely high fecundity; small eggs	
Longfin Eel	*Anguilla dieffenbachii*	Age at maturity (×2)	3	Very late maturing (> 25 years males, > 40 years females)	
Longfin Eel	*Anguilla dieffenbachii*	Dispersal/recruitment (×2)	1	Catadromous with wide dispersal	
Longfin Eel	*Anguilla dieffenbachii*	Adult body size (×2)	1	Can exceed 1 m; not susceptible to trout predation	
Shortfin Eel	*Anguilla australis*	Habitat overlap	2	Coexists in lowland habitats with trout	Beentjes et al. (2006); Cadwallader (1975a, 1975b); Dunn et al. (2018); Glova et al. (1998); Jellyman et al. (2013); Jowett and Richardson (1995); McDowall (1990); Ryan (1986); Sagar and Glova (1998)
Shortfin Eel	*Anguilla australis*	Diet similarity	2	Carnivorous; diet overlaps with trout	
Shortfin Eel	*Anguilla australis*	Diel activity	1	Nocturnal; avoids trout activity peaks	
Shortfin Eel	*Anguilla australis*	Fecundity & egg size (×2)	1	High fecundity; small eggs	
Shortfin Eel	*Anguilla australis*	Age at maturity (×2)	2	Matures > 14 years (males)	
Shortfin Eel	*Anguilla australis*	Dispersal/recruitment (×2)	1	Catadromous and widespread	
Shortfin Eel	*Anguilla australis*	Adult body size (×2)	1	Large; unlikely to be predated by trout	
Common bully	*Gobiomorphus cotidianus*	Habitat overlap	2	Ubiquitous in lowland waters with trout	Closs et al. (2003); David et al. (2002); Dunn et al. (2018); Jellyman et al. (2013); Jowett and Richardson (1995); McDowall (1990); West, David, Closs, et al. (2015)
Common bully	*Gobiomorphus cotidianus*	Diet similarity	2	Feeds on benthic invertebrates	
Common bully	*Gobiomorphus cotidianus*	Diel activity	2	Active during day and night; overlaps with trout	
Common bully	*Gobiomorphus cotidianus*	Fecundity & egg size (×2)	1	Moderate fecundity	
Common bully	*Gobiomorphus cotidianus*	Age at maturity (×2)	1	Matures at 1–2 years	
Common bully	*Gobiomorphus cotidianus*	Dispersal/recruitment (×2)	1	Widespread dispersal; estuarine links	
Common bully	*Gobiomorphus cotidianus*	Adult body size (×2)	2	Typical adult ~90 mm	
Giant bully	*Gobiomorphus gobioides*	Habitat overlap	2	Lower rivers and estuaries with trout	Dunn et al. (2018); Jellyman et al. (2000); Jellyman et al. (2013); Ling, West, et al. (2015); McDowall (1990); McDowall (1997)
Giant bully	*Gobiomorphus gobioides*	Diet similarity	2	Eats small fish and inverts; some overlap	
Giant bully	*Gobiomorphus gobioides*	Diel activity	2	Day and crepuscular; overlaps with trout	
Giant bully	*Gobiomorphus gobioides*	Fecundity & egg size (×2)	1	High fecundity	
Giant bully	*Gobiomorphus gobioides*	Age at maturity (×2)	2	Matures at 2–3 years	
Giant bully	*Gobiomorphus gobioides*	Dispersal/recruitment (×2)	1	Amphidromous; broad dispersal	
Giant bully	*Gobiomorphus gobioides*	Adult body size (×2)	1	Large body size reduces trout vulnerability	
Black Flounder	*Rhombosolea retiaria*	Habitat overlap	2	Inhabits estuaries and lower rivers with trout	David, Franklin, et al. (2015); Dunn et al. (2018); Jellyman et al. (2013); McDowall (1990); McDowall (2016); Waimaori (2017)
Black Flounder	*Rhombosolea retiaria*	Diet similarity	2	Feeds on benthic inverts and whitebait, shared with trout	
Black Flounder	*Rhombosolea retiaria*	Diel activity	1	Benthic and cryptic; minimal interaction	
Black Flounder	*Rhombosolea retiaria*	Fecundity & egg size (×2)	1	High fecundity, marine spawning	
Black Flounder	*Rhombosolea retiaria*	Age at maturity (×2)	2	Late onset of breeding age	
Black Flounder	*Rhombosolea retiaria*	Dispersal/recruitment (×2)	1	Marine dispersal phase	
Black Flounder	*Rhombosolea retiaria*	Adult body size (×2)	1	Too large and benthic for trout predation	
Pouched Lamprey	*Geotria australis*	Habitat overlap	1	Adults and ammocoetes occupy deep pools or sediment; limited trout overlap	Closs et al. (2015); Dunn et al. (2018); James (2008); Jellyman and Glova (2002); Jellyman et al. (2002); Jellyman et al. (2013); Jowett et al. (1996); Kelso and Glova (1993); McDowall (1990); Paton et al. (2019)
Pouched Lamprey	*Geotria australis*	Diet similarity	1	No feeding in freshwater; no overlap	
Pouched Lamprey	*Geotria australis*	Diel activity	1	Strictly nocturnal	
Pouched Lamprey	*Geotria australis*	Fecundity & egg size (×2)	1	High fecundity	
Pouched Lamprey	*Geotria australis*	Age at maturity (×2)	3	Long larval period (~4 years)	
Pouched Lamprey	*Geotria australis*	Dispersal/recruitment (×2)	1	Anadromous; widespread dispersal	
Pouched Lamprey	*Geotria australis*	Adult body size (×2)	1	Adults are too large for trout to prey on	
